# Objectively Quantifying Pediatric Psychiatric Severity Using Artificial Intelligence, Voice Recognition Technology, and Universal Emotions: Pilot Study for Artificial Intelligence-Enabled Innovation to Address Youth Mental Health Crisis

**DOI:** 10.2196/51912

**Published:** 2023-10-23

**Authors:** Desmond Caulley, Yared Alemu, Sedara Burson, Elizabeth Cárdenas Bautista, Girmaw Abebe Tadesse, Christopher Kottmyer, Laurent Aeschbach, Bryan Cheungvivatpant, Emre Sezgin

**Affiliations:** 1 School of Electrical and Computer Engineering Georgia Institute of Technology Atlanta, GA United States; 2 TQIntelligence, Inc Atlanta, GA United States; 3 Department of Psychiatry and Behavioral Sciences, Computational Psych Program Morehouse School of Medicine Atlanta, GA United States; 4 Microsoft AI for Good Research Lab Nairobi Kenya; 5 Abigail Wexner Research Institute Nationwide Children's Hospital Columbus, OH United States

**Keywords:** pediatric, trauma, voice AI, machine learning, mental health, predictive modeling, artificial intelligence, social determinants of health, speech-recognition, adverse childhood experiences, trauma and emotional distress, voice marker, speech biomarker, pediatrics, at-risk youth

## Abstract

**Background:**

Providing Psychotherapy, particularly for youth, is a pressing challenge in the health care system. Traditional methods are resource-intensive, and there is a need for objective benchmarks to guide therapeutic interventions. Automated emotion detection from speech, using artificial intelligence, presents an emerging approach to address these challenges. Speech can carry vital information about emotional states, which can be used to improve mental health care services, especially when the person is suffering.

**Objective:**

This study aims to develop and evaluate automated methods for detecting the intensity of emotions (anger, fear, sadness, and happiness) in audio recordings of patients’ speech. We also demonstrate the viability of deploying the models. Our model was validated in a previous publication by Alemu et al with limited voice samples. This follow-up study used significantly more voice samples to validate the previous model.

**Methods:**

We used audio recordings of patients, specifically children with high adverse childhood experience (ACE) scores; the average ACE score was 5 or higher, at the highest risk for chronic disease and social or emotional problems; only 1 in 6 have a score of 4 or above. The patients’ structured voice sample was collected by reading a fixed script. In total, 4 highly trained therapists classified audio segments based on a scoring process of 4 emotions and their intensity levels for each of the 4 different emotions. We experimented with various preprocessing methods, including denoising, voice-activity detection, and diarization. Additionally, we explored various model architectures, including convolutional neural networks (CNNs) and transformers. We trained emotion-specific transformer-based models and a generalized CNN-based model to predict emotion intensities.

**Results:**

The emotion-specific transformer-based model achieved a test-set precision and recall of 86% and 79%, respectively, for binary emotional intensity classification (high or low). In contrast, the CNN-based model, generalized to predict the intensity of 4 different emotions, achieved test-set precision and recall of 83% for each.

**Conclusions:**

Automated emotion detection from patients’ speech using artificial intelligence models is found to be feasible, leading to a high level of accuracy. The transformer-based model exhibited better performance in emotion-specific detection, while the CNN-based model showed promise in generalized emotion detection. These models can serve as valuable decision-support tools for pediatricians and mental health providers to triage youth to appropriate levels of mental health care services.

**International Registered Report Identifier (IRRID):**

RR1-10.2196/51912

## Introduction

### Background

The mental health care delivery system of care for youth is in crisis. The suicide rate for youths aged 10 to 24 years increased by 62% between 2007 and 2021 [1]. There is a shortage of trained mental health professionals, especially in low- and middle-income countries and communities, having less than one mental health professional per 10,000 people [2]. Training professionals and therapists require comprehensive effort to develop the appropriate levels of clinical skills equal to the trauma-based severe mental health issues in these communities. Conventional training methods, which typically involve long durations of apprenticeship and supervision, are often resource-intensive. The current system struggles to meet the growing demand for mental health services, especially for pediatric patients and youths [3]. Furthermore, evaluating the effectiveness of therapy remains challenging, as mental health conditions are often complex and multifaceted, and current evaluation methods primarily rely on subjective assessments [4].

The use of artificial intelligence (AI) in emotion detection from audio recordings is an emerging approach to address these challenges. Studies have shown that speech carries rich information beyond words, including vital emotional cues and biomarkers associated with mental health conditions [5]. This helps in measuring the effectiveness of therapy and identifying patients in high-risk environments, thus enabling timely interventions. Such quantifying emotions also aids in the optimal allocation of resources by preventing unnecessary treatments [6].

In this paper, we present the development of a deep learning model that classifies emotions using a 15- to 90-second voice sample collected as part of a counseling session. Our study focuses on audio recordings of children with significant psychiatric symptoms partly related to high adverse childhood experience (ACE) scores reading a fixed script. In total, 4 highly trained therapists classified audio segments based on 1 of the 4 emotion intensity levels (none, low, medium, and high) for each of the 4 emotions (anger, fear, happiness, and sadness). The paper reports the development and training of the machine learning (ML) models and discusses the implications of our findings for the broader landscape of mental health care.

As surveyed by Koolagudi and Rao [7], speech emotion recognition (SER) is a prevalent area of research with numerous classification models, feature extractors, and publicly available data sets. However, there are many limitations in the current body of research. Many corpus of speech data are focused on recognizing actor-simulated speech emotion, which contains much higher emotional intensities than what is found in ordinary speech. Additionally, many research results are based on data sets with a limited number of speakers. For example, the IEMOCAP data set [8] is frequently used in literature for SER tasks. However, it comprises actor-simulated emotional utterances from only 10 different speakers. It is also noteworthy that classiﬁcation of the intensity of emotion found in speech is much more limited. The RAVDESS data set is one of a few publicly available data sets containing actor-simulated audio recordings labeled with emotion intensity (either low or high) [9]. The data sets found in the literature are also very short in duration. The audio clips in IEMOCAP and RAVDESS are both segmented at the utterance level, such that one audio clip only contains a handful of spoken words and is usually only 1-2 seconds in duration.

Numerous model architectures have been used for SER tasks. Zhang et al [10] developed a fully convolutional network (FCN) to classify the emotion type from audio recordings in the IEMOCAP data set [8]. They used an Alexnet-based [11] convolutional neural network (CNN) for feature extraction and a soft-attention–based classiﬁer, resulting in a class-weighted accuracy of around 68%. This approach was appealing to us due to its insensitivity to input length the FCN is able to handle inputs of different sizes without the need to crop or divide inputs into multiple segments.

### Participants

Participants were recruited from 3 mental health care centers that focused primarily on children and adolescents from low-income communities. The participants receive mental health services paid for by Medicaid, the Children’s Health Institute Program, and the Department of Children and Family Services. The participants are children aged 5-18 years and those referred for behavioral health services through schools, DJJ, and child protective agencies.

## Methods

### Data Exploration and Processing

The data set used for this project was gathered as part of the National Science Foundation Small Business Innovation Research Phase I and Phase II study. It encompassed 1055 audio recordings obtained during therapy sessions with children who are receiving mental health services and who have been diagnosed with at least 1 DSM (Diagnostic and Statistical Manual of Mental Disorders) condition. The recordings focused on the children reading scripted passages, though the textual content itself was not a central aspect of the project. Each audio recording was meticulously annotated with an emotional intensity level (none, low, medium, or high) for 4 emotions, namely anger, fear, sadness, and happiness. To ensure robustness, 4 different labelers were involved in this annotation process for each recording, and the attribution of each labeler was recorded.

Throughout the exploratory phase, several noteworthy observations were made regarding the data. One key finding was that meaningful disagreements among the labelers were evident due to multiple labelers providing annotations for most recordings. Additionally, the recordings exhibited a degree of noise, with varying background noise levels, the occasional presence of multiple speakers (such as the therapist themselves), and instances where the speaker sounded distant from the recording device. Moreover, the duration of the recordings varied significantly, ranging from as short as 2 seconds to over 6 minutes. Though the majority fell within the 30-90 seconds range, this presented a notable difference compared to typical data sets found in the existing literature.

A contributing factor to these challenges can be attributed to the data collection process, which took place in the natural environments of the patients, such as their schools or homes. While this approach offers valuable insights, it also introduced certain complexities that needed to be addressed during data processing and analysis.

### Data Preprocessing

#### Label Disagreement

Various methodologies were used to address the discrepancies among labelers in handling emotion intensities. First, we transitioned from a 4-class classification to a binary classification, distinguishing between low and high emotion intensities. To achieve this, we combined the categories “none” and “low” to represent the binary class 0, and similarly, we grouped “medium” and “high” for the binary class. Additionally, we trained 2 distinct types of models: one where the mode predictions across the 4 labelers served as the “ground truth,” and another where we developed individual models for each labeler. This approach allowed us to explore different perspectives and optimize model performance.

#### Denoising

As stated earlier, some of the voice samples were noisy despite the therapist’s effort to manage the background noise. Although some of the models used have noise resistance capabilities, it was often not sufficient for the models to achieve good results. Therefore, data set denoising had to be carried out. In recent years, neural network denoising has become the state-of-the-art approach to solving speech denoising. Therefore, one of the latest works [3] from Facebook was applied. It uses Demucs architecture, which was originally developed for the task of music source separation [12].

#### Voice Activity Detection

The data set’s recordings include extended periods of silence and background noise with no speech. When training models, especially those with attention layers, using extensive portions of audio from silent regions leads to less effective results, and the training process takes longer. This issue is partly influenced by participants experiencing severe symptoms of depression or anxiety, requiring substantial support to finish the reading tasks.

To address this, we conducted an experiment where we applied a voice activity detection (VAD) algorithm to identify and remove sections of the audio files where no active speaker was present. For this purpose, we used Silero models to extract these voice fragments [13].

#### Diarization

Another issue with the data set is the presence of various voices besides those of children, such as therapists asking questions, among others. The inclusion of these additional voices can lead the model to focus on irrelevant parts of the input data, resulting in poorer performance. To address this problem, we conducted experiments involving a diarization algorithm to process the data, retaining only the segments with children’s voices. Diarization models, however, cannot definitively identify child speakers. Nevertheless, in recordings where strangers speak, children’s voices generally dominate in terms of time, making it possible to distinguish and isolate them. It is important to mention that certain recordings experienced high levels of noise, leading to instances where the diarization algorithm detected 3 or more speakers, particularly in shorter audio recordings. Consequently, we decided to filter out such instances from our data set.

### Modeling

#### Fully Convolutional Networks

In recent years, FCNs have emerged as a favored method for speech-emotion recognition due to their modest parameter count and ability to handle variable-length inputs. In this study, we developed FCN models based on the work of [10].

To begin, we computed the Mel spectrogram, which captures both frequency and intensity variations in the audio clip over time, representing it as a single image per recording. The advantage of using FCNs is that they can directly process these variable-length images without the need for resizing or cropping. Longer audio clips produce larger spectrograms, but the FCN efficiently handles them.

Subsequently, the image was fed through a CNN to extract meaningful features from the spectrogram, facilitating emotion intensity classification. Like how individual consonant and vowel sounds can be discerned from inspecting the spectrogram, we posit that comparable features exist for emotion intensity identification.

Finally, the outputs of the CNN are passed through one or more parallel output blocks, each comprising a 1×1 convolution step followed by a global-average pooling layer. This step reduces the dimensionality to the number of emotion intensity classes, independent of the input size. Consequently, classification is achieved without the need for dense layers with fixed dimensions.

We performed fine-tuning on 2 pretrained CNN backbones: Alexnet [11] and Googlenet [14]. Both these architectures are well-known CNNs that were pretrained on ImageNet and have been widely used for various image classification tasks [15,16]. We chose Alexnet for its compact size and previous success in similar speech-emotion recognition tasks in the literature. On the other hand, Googlenet was selected for its use of “inception blocks,” which we believed might offer advantages with its convolutional filters of different sizes. To adapt these pretrained networks, we worked with their Pytorch implementations.

Our experimentation explored 2 output layer structures: “single-head” and “multi-head.” The models with a single output block enabled the prediction of emotion intensity for one specific emotion, while the multi-head model consisted of 4 separate output blocks, one for each emotion. Although a multi-head model would be more convenient, we aimed to investigate whether there were any performance differences in learning features for classifying a single emotion compared to learning features that could classify all 4 emotions simultaneously.

#### Speechbrain

All models described earlier require a Mel spectrogram as an input. However, this speech representation has disadvantages, such as loss of information, complexity, etc. It also requires additional model capacity to be trained to extract useful information for each specific task. That is why, in recent times, self-supervised models for speech processing have gained a lot of attention. As can be deduced by its name, these models are trained in a self-supervised fashion like popular Natural Language Processing models such as BERT. The most popular ones are Hubert [17] and Wav2Vec 2.0 [18]. Both models have convolution layers in the beginning and Transformer layers after the convolution layers. The high-level pipeline is similar: the model accepts raw input audio and is trained to predict the cluster number for each audio chunk.

There are, however, some cluster refinement differences between the models. This enables them to extract phoneme-like structures in audio data and helps to extract more useful information, especially for speech-processing tasks. It is important to note that the model has some denoising capabilities by design, as this pipeline resembles the one used to train Automatic speech recognition models. The major difference is that instead of phonemes, we have cluster numbers. These types of models are implemented in a framework called Speech-the brain. In this work, we used architecture Wav2Vec 2.0. To apply this model to our task, its’ architecture needed to be slightly modified and fine-tuned. We added a final head as a multilayer perceptron layer after the transformer modules. The model was trained in the usual classification problem manner with MSE loss. The gradient was backpropagated to all Wav2Vec2.0 layers. Each model was trained for 30 epochs. The multilayer perceptron layer has 2 outputs, and the model is trained to differentiate whether an emotion appears in the input or not. The setup with classifying all emotions at once was also attempted but gave poor results compared to a binary classifier.

### Ethical Considerations

The study received ethical board approval (protocol #21-TQIN-101) for a multisite pilot involving children and adolescents. We will receive written and informed consent from participants. The activities for patients enrolled in the study include data collected using multiple surveys and a voice sample. The surveys include structured and unstructured voice samples based on a reading selected by the research team. The ethical board approves all the study contents.

### Data Collection

ThinkQuality, TQI’s Phone App, was used to collect voice samples from youth receiving mental health and family preservation services at the point of care. The data collection process includes concurrently administering a scientifically validated survey. The patient’s emotional disorder severity is confirmed based on the scores of the Symptoms and Functioning Severity Scale (SFSS), ACE, and Patient Health Questionnaire-9 (PHQ-9), the patient’s diagnosis, and other clinical information from the patient’s treatment history. For every voice sample collected, the pilot has a diagnosis or multiple, current procedural terminology code, and the therapist’s clinical input. These data serve to categorize voice sample data into distinct categories and train voice-based ML algorithms to distinguish children in need and at high risk. The innovation is focused on families with low socioeconomic status, in which ACEs are common.

Data are collected by therapists (n=35). An Android mobile app collects speech samples, SFSS, PHQ-9, and ACE scale responses. Each therapist receives a 3 to 5-hour in-person training about app installation and access, data (SFSS, voice sample) collection using the app, compliance and parameters, the process of the reward program, communication, and real-time technical support. Data are collected from patients during mental health or other family-based intervention visits weekly or biweekly, based on the intensity of the psychiatric symptoms. Visits occur in a suitable (private) location at home (including foster and group homes) or school as part of a state’s commitment to reduce barriers to services and easily access services. Data are collected partially web-based during the service shift secondary to the COVID-19 pandemic.

To elicit speech for recording and analysis, patients are asked to choose from a subset of readings. Patients’ reading is recorded for up to 90 seconds—a clock feature in the app signals when the time is up. In general, if the child’s reading label is like the grade, the reading could be completed in less than 50 seconds. Voice samples are stored in a separate deidentified database, allowing 3 to 4 psychologists to label the voice sample independently. SFSS, PHQ-9, and ACE responses are collected using the same app. The app scores the surveys immediately and makes the results available to the therapist to share with their patients if it is clinically appropriate; real-time data availability is intended to close the gap in transparency, accountability, and family engagement.

## Results

### Overview

The primary goal of this research was to investigate diverse data preprocessing and modeling approaches in order to identify fruitful opportunities. As a result, significant dedication was put into testing various combinations within our suggested methodology to discover promising options. To facilitate this process, we extensively used the experiment tracking platform called Biases. It enabled us to store training results and test metrics, and save potential model candidates for future deployment on the web app. While most models underwent 10 epochs of training with a learning rate of 5e-5, a few were terminated earlier due to the validation loss plateauing without any additional performance gains.

### Evaluation Metrics

The evaluation process for the FCN models involved computing metrics using a 15% hold-out test set. Conversely, for the speech brain-based models, metrics were computed using a 10% hold-out test set. Since the task at hand was a binary classification problem, we used precision and recall as our evaluation metrics. The primary objective was to identify a model capable of accurately detecting high intensity of emotion within audio files while keeping false positives to a minimum.

### Denoising

First, the denoising effects were evaluated by initially applying a pretrained denoiser model to the unmodified source audio files as a preprocessing step. Subsequently, 3 pairs of models were trained, each corresponding to 1 of 3 emotions (anger, fear, and sadness). Each pair included 1 model trained on the original audio files and another model trained on the audio files with the denoiser applied.

To maintain consistency, only the labels from a single labeler (Labeler 1) were used, given their extensive number of labeled samples. Additionally, a similar pair was trained using the model with all 4 emotion heads. The results of these experiments can be found in Table 1.

The outcomes revealed that denoising alone led to a slight improvement in the Anger emotion, but resulted in a marginal or more significant performance decrease for the FCN model in the other emotions. Moreover, the FCN model equipped with 4 output heads exhibited worse performance when denoising was added.

**Table 1:** Fully convolutional network model results with and without denoiser for audio samples labeled by Labeler 1.

Emotion and denoise			Precision	Recall
**Anger**				
	No	0.612		0.513
	Yes	0.656		0.667
**Fear**				
	No	0.741		0.769
	Yes	0.717		0.756
**Sadness**				
	No	0.801		0.801
	Yes	0.667		0.737
**All 4**				
	No	0.833		0.833
	Yes	0.806		0.801

### VAD

The impact of VAD on the results was analyzed through a series of experiments. Initially, the unmodified source audio files underwent preprocessing with a pretrained VAD model. In total, 3 sets of emotion-specific models were then trained, both with and without the application of VAD. Additionally, a model with all 4 emotion heads was trained using VAD. The outcomes are presented in Table 2. It was observed that applying VAD led to negligible or marginal enhancements in the single-emotion FCN models’ performance while causing a decline in the performance of the 4-emotion FCN model.

**Table 2:** Fully convolutional network model results with and without VAD^a^ for audio samples labeled by Labeler 1.

Emotion and VAD		Precision	Recall
**Anger**			
	No	0.612	0.513
	Yes	0.627	0.609
**Fear**			
	No	0.741	0.769
	Yes	0.729	0.763
**Sadness**			
	No	0.801	0.801
	Yes	0.808	0.808
**All 4**			
	No	0.833	0.833
	Yes	0.814	0.793

^a^VAD: voice activity detection.

### Denoising+VAD

The effects of preprocessing with both the denoiser and VAD were similarly compared. In this case, the unmodified source audio files first had the denoiser applied, followed by VAD. Then, the same 3 pairs of models, one per emotion, were trained, as well as the pair using all 4 emotion heads. The results are shown in Table 3.

The effects of preprocessing with the denoiser and VAD were also compared against a baseline using only VAD (rather than a baseline without any preprocessing). This set of models was trained using the speech brain-based model. The results are shown in Table 4. As we can see, in some cases, denoising boosts the quality, but in some cases, it decreases quality. In cases where quality decreased, the primary cause was extremely noisy data, and the denoiser overcompensated.

**Table 3:** Fully convolutional network model results with and without both denoiser and VAD^a^ combined for audio samples labeled by Labeler 1.

Emotion and denoise+VAD		Precision	Recall
**Anger**			
	No	0.612	0.513
	Yes	0.651	0.550
**Fear**			
	No	0.741	0.769
	Yes	0.761	0.776
**Sadness**			
	No	0.801	0.801
	Yes	0.711	0.756
**All 4**			
	No	0.833	0.833
	Yes	0.803	0.792

^a^VAD: voice activity detection.

**Table 4:** Speechbrain model results with and without denoiser for audio samples with voice activity detection applied and labeled by Labeler 1.

Emotion and denoise		Precision	Recall
**Anger**			
	No	0.777	0.734
	Yes	0.783	0.765
**Fear**			
	No	0.811	0.82
	Yes	0.812	0.845
**Sadness**			
	No	0.781	0.81
	Yes	0.863	0.794

### Denoising+VAD+Diarization

Experiments with diarization were also conducted. It can be observed that for a particular labeler, a slight decrease in performance occurred, which indicates overwork. The results are shown in Table S5 in Multimedia Appendix 1.

### Alexnet Versus Googlenet

Another comparison we were interested in is how different CNN architectures performed on this data set. Alexnet and Googlenet are both powerful backbones used in similar image recognition tasks. To compare the two, we trained 2 models—1 pair with only a single prediction head (for the fear emotion) while the other pair had 4 prediction heads, 1 for each emotion. These models were trained without any preprocessing steps applied to the audio files. Unfortunately, not many experiments could be run on this combination due to the long training time required for Googlenet (10-15 times slower than Alexnet). Overall, Googlenet performed worse in both cases than Alexnet. Part of this could be attributed to a much trickier training process for Googlenet—it was difficult to dial the learning rate to prevent the model from over-fitting after only 1-2 epochs. The results are shown in Table S6 in Multimedia Appendix 2.

### Single- and Multi-Head Models

In our study, we conducted a comparison between models equipped with single and multiple output heads. To achieve this, we trained a model using 4 output heads to predict emotion intensity for 4 emotions. This training used data from a single labeler. Subsequently, we used the same labeled data to train 4 separate models, each with only 1 output head dedicated to predicting a specific emotion. Both Labeler 1 and Labeler 3 data sets were used for this purpose.

Although direct comparison of the results proved challenging, they provided valuable insights. The model with multiple output heads demonstrated reasonable performance and merits further exploration. It is possible that optimizing the model with multiple emotion heads simultaneously may lead to intermediate features that are more generalized, enhancing the prediction of various emotions. For detailed results, please refer to Table S7 in Multimedia Appendix 3.

### One Model per Emotion

In the preceding sections, the focus was solely on models trained with data from individual speakers. However, it is more practical to use 1 model per emotion during the inference process. To explore this approach, several experiments were conducted, and the outcomes are presented in Table S8 in Multimedia Appendix 4. For these experiments, all 3 preprocessing techniques (denoising, VAD, and diarization) were applied.

The results obtained from this approach were not as promising as those achieved with separate labelers, mainly due to the lack of consensus among data labelers. This was further confirmed by computing the Krippendorf coefficient for various subsets of labelers, as shown in Table S1 in Multimedia Appendix 5. The majority of combinations yielded results close to zero, indicating a significant lack of agreement among speakers. However, it is worth noting that for the emotion “sadness,” some combinations did achieve a score of 0.45, which is considered acceptable.

### FCN Versus Speechbrain

It may also be useful to compare the differences in model architectures. For this experiment, models were trained on a per-labeler, per-emotion basis using both the Alexnet-based FCN model and the Speechbrain model. The preprocessing was consistent in this comparison, with both models using the data set with denoising and VAD applied. Attention-based models like Wav2vec 2.0 can determine temporal relationships, dependencies, and correlations that are not linked with a local aspect like CNN or sequential like an RNN. The wav2vec 2.0 model can capture long-term temporal dependencies from time stamp complexity. Since the audio files contain multiple emotions and are quite long, this explains why wav2vec 2.0 outperforms the FCN-based model. The results are shown in Table 5.

**Table 5:** Comparing FCN^a^ and Speechbrain models using data with denoiser, and voice activity detection applied.

Labeler and emotion		Precision (FCN)	Precision (Speechbrain)	Recall (FCN)	Recall (Speechbrain)
**Labeler 1**					
	Anger	0.651	0.782	0.660	0.765
	Fear	0.761	0.811	0.776	0.844
	Sadness	0.711	0.862	0.756	0.794
**Labeler 3**					
	Anger	0.670	0.818	0.690	0.823
	Fear	0.715	0.811	0.671	0.785
	Sadness	0.723	0.845	0.723	0.846
**Labeler 2**					
	Anger	0.934	0.832	0.966	0.832
	Fear	0.677	0.853	0.685	0.853
	Sadness	0.684	0.885	0.685	0.882
**Labeler 4**					
	Anger	0.742	0.837	0.739	0.842
	Fear	0.658	0.781	0.640	0.755
	Sadness	0.841	0.835	0.802	0.763

^a^FCN: fully convolutional network.

## Discussion

### Principal Findings

In this study, we developed a deep learning model to classify emotions from voice samples collected during the psychotherapy session. Audio recordings from a clinical population have been considered a critical step in leveraging AI-driven tools for mental health care. These models can serve as valuable decision-support tools for pediatricians and mental health providers to triage youth to appropriate levels of mental health care services. The ability to accurately quantify emotional states can potentially improve disparities in treatment outcomes, especially for youth from low-income communities and marginalized communities. Our model demonstrated proficiency in predicting therapists’ intensity-based labels for different emotions.

There are several implications and potential applications of this proposed model. First, the ability to objectively measure emotional states could complement therapists’ subjective assessments, leading to more precise and data-driven clinical decision-making in therapeutic interventions. In addition, quantifying emotions can contribute to streamlining resource allocation in mental health care, ensuring that individuals in critical need receive timely attention; the crisis in youth mental health and projected shortage of qualified mental health providers may require AI-driven solutions to effectively manage resources by focusing on the neediest.

However, the implementation of such models should regard ethical considerations and adoption. Patient privacy and data security must be ensured. Moreover, as AI can significantly augment the capabilities of professionals, it should not be introduced as a replacement for human connection, which is at the root of effective psychotherapeutic endeavors; instead, it should be used as a supporting tool that enhances therapists’ abilities to make informed decisions.

### Limitations

The study presents several limitations. The quality of speech data, including background noise and speech disorders, continues to affect the accuracy and reliability of the ML model. Second, the sample population may not be representative of the greater US population, limiting the generalizability of findings; participants have at least 1 DSM diagnosis. There are very limited voice samples of happiness. Finally, legal and regulatory considerations, including privacy rules and compliance with regulations, must be reviewed based on the state and jurisdictions and may limit the use of the algorithm. The latter must be considered to protect participants and ensure the lawful use of data [19].

### Implication and Future Work

We discovered that many of our models, including our best one, hit some kind of predictive ceiling. We believe data quality may be the limiting factor in this case. Since the data labels apply to long audio segments, information density is relatively low. It is difficult to know whether a specific utterance in a long recording caused a labeler to label the audio anger as high or if the overall tone of the speaker throughout the recording indicated a high anger level. One valuable area for future work would be to break down the audio clips into smaller chunks, perhaps even to the sentence or utterance level. Having more granular labels would enable their data scientists to incorporate commonly used data sets found in literature as additional training samples. We have started breaking down the length of the audio to 15 seconds and labeling them; accordingly, we plan to publish the result of this process.

Another area of potential future work is to work on model interpretability. Tools like saliency maps are commonly used in vision problems to understand better the features extracted at intermediate model layers. Trying similar techniques to help diagnose the current model limitations would be interesting. Perhaps this could be extended to reconstructing the relevant audio segments from the salient portions of the spectrogram and see if the labelers agree with what the model picks up on.

### Conclusions

The children and adolescent system of care remains volatile despite the end of the COVID-19 pandemic that precipitated the current crisis; rural and intercity locations have taken the brunt of symptom severity and lack of access. The integration of digital mental health innovations is crucial in addressing the ongoing mental health crisis; these technologies need to fit into the existing workflow of medical providers and therapists. Speech-based digital biomarkers that can be collected quickly via a user-friendly interface hold promise for identifying emotional distress and functional impairments in this population. This study is one such technology that contributes to the broader digital health transformation and paves the way for proactive and collaborative mental health care services to improve treatment outcomes and reduce disparities for individuals from low-income communities and marginalized groups.

## Appendix

Multimedia Appendix 1 Speechbrain model results with and without diarization for audio samples labeled by Labeler 1.

Multimedia Appendix 2 Fully convolutional network model results comparing Alexnet and Googlenet for audio samples labeled by Labeler 1.

Multimedia Appendix 3 Fully convolutional network model results comparing Single and Multiple output heads for Labeler 1 and Labeler 3.

Multimedia Appendix 4 Speechbrain model results for aggregated labels.

Multimedia Appendix 5 Krippendorf metrics for all combinations of labelers.

Multimedia Appendix 6 Peer review reports.

## Abbreviations

ACE: adverse childhood experience
AI: artificial intelligence
CNN: convolutional neural network
DSM: Diagnostic and Statistical Manual of Mental Disorders
FCN: fully convolutional network
ML: machine learning
PHQ-9: Patient Health Questionnaire-9
SER: speech emotion recognition
SFSS: Symptoms and Functioning Severity Scale
VAD: voice activity detection

## Data Availability

Data sharing is not applicable to this study as no data sets were generated or analyzed during this study. Preliminary work was completed using a public data set, and the data set is cited in the paper.
