# Supplementary material for: Objectively Quantifying Pediatric Psychiatric Severity Using Artificial Intelligence, Voice Recognition Technology, and Universal Emotions: Pilot Study for Artificial Intelligence-Enabled Innovation to Address Youth Mental Health Crisis
Source: JMIR Res Protoc. 2023 Oct 23;12:e51912. doi: 10.2196/51912 (PMC10628686; doi:10.2196/51912)
Supplement: Multimedia Appendix 1 [file resprot_v12i1e51912_app1.docx]

**Multimedia Appendix 1**

**Table S5.** Speechbrain model results with and without diarization for audio samples labeled by Labeler 1.

| Emotion | Diarization | Precision | Recall |
| --- | --- | --- | --- |
| Anger | Yes | 0.792 | 0.7315 |
| Fear | Yes | 0.822 | 0.842 |
| Sadness | Yes | 0.871 | 0.783 |
