# Supplementary material for: Objectively Quantifying Pediatric Psychiatric Severity Using Artificial Intelligence, Voice Recognition Technology, and Universal Emotions: Pilot Study for Artificial Intelligence-Enabled Innovation to Address Youth Mental Health Crisis
Source: JMIR Res Protoc. 2023 Oct 23;12:e51912. doi: 10.2196/51912 (PMC10628686; doi:10.2196/51912)
Supplement: Multimedia Appendix 2 [file resprot_v12i1e51912_app2.docx]

**Multimedia Appendix 2**

**Table S6.** Fully convolutional network model results comparing Alexnet and Googlenet for audio samples labeled by Labeler 1.

| Emotion | Backbone | Precision | Recall |
| --- | --- | --- | --- |
| **Fear** | | | |
|  | Alexnet | 0.741 | 0.769 |
|  | Googlenet | 0.720 | 0.718 |
| **All 4** | | | |
|  | Alexnet | 0.833 | 0.833 |
|  | Googlenet | 0.704 | 0.694 |
