# Supplementary material for: Objectively Quantifying Pediatric Psychiatric Severity Using Artificial Intelligence, Voice Recognition Technology, and Universal Emotions: Pilot Study for Artificial Intelligence-Enabled Innovation to Address Youth Mental Health Crisis
Source: JMIR Res Protoc. 2023 Oct 23;12:e51912. doi: 10.2196/51912 (PMC10628686; doi:10.2196/51912)
Supplement: Multimedia Appendix 3 [file resprot_v12i1e51912_app3.docx]

**Multimedia Appendix 3**

**Table S7.** Fully convolutional network model results comparing Single and Multiple output heads for Labeler 1 and Labeler 3.

| Labeler | Emotion | Precision | Recall |
| --- | --- | --- | --- |
| **YA** | | | |
|  | Anger | 0.612 | 0.513 |
|  | Fear | 0.740 | 0.769 |
|  | Happy | 1.00 | 1.00 |
|  | Sadness | 0.801 | 0.801 |
|  | All 4 | 0.833 | 0.833 |
| **Machine Learning** | | | |
|  | Anger | 0.710 | 0.677 |
|  | Fear | 0.669 | 0.619 |
|  | Happy | 0.900 | 0.948 |
|  | Sadness | 0.740 | 0.736 |
|  | All 4 | 0.757 | 0.763 |
