# Supplementary material for: Objectively Quantifying Pediatric Psychiatric Severity Using Artificial Intelligence, Voice Recognition Technology, and Universal Emotions: Pilot Study for Artificial Intelligence-Enabled Innovation to Address Youth Mental Health Crisis
Source: JMIR Res Protoc. 2023 Oct 23;12:e51912. doi: 10.2196/51912 (PMC10628686; doi:10.2196/51912)
Supplement: Multimedia Appendix 4 [file resprot_v12i1e51912_app4.docx]

**Multimedia Appendix 4**

**Table S8.** Speechbrain model results for aggregated labels.

| Emotion | Precision | Recall |
| --- | --- | --- |
| Anger | 0.839 | 0.765 |
| Fear | 0.788 | 0.802 |
| Sadness | 0.762 | 0.733 |
