# Supplementary material for: Objectively Quantifying Pediatric Psychiatric Severity Using Artificial Intelligence, Voice Recognition Technology, and Universal Emotions: Pilot Study for Artificial Intelligence-Enabled Innovation to Address Youth Mental Health Crisis
Source: JMIR Res Protoc. 2023 Oct 23;12:e51912. doi: 10.2196/51912 (PMC10628686; doi:10.2196/51912)
Supplement: Multimedia Appendix 5 [file resprot_v12i1e51912_app5.docx]

# Multimedia Appendix

**Table S1.** Krippendorf metrics for all combinations of labelers.

| Therapists | Anger | Happy | Sadness | Fear |
| --- | --- | --- | --- | --- |
| Labeler 3, Labeler 2 | –0.1377 | 0.1755 | 0.4530 | –0.0278 |
| Labeler 3, Labeler 4 | 0.2662 | 0.0103 | 0.0742 | 0.0710 |
| Labeler 3, Labeler 1 | 0.1183 | –0.0230 | 0.2422 | 0.1169 |
| Labeler 2, Labeler 4 | –0.1264 | –0.0033 | 0.1074 | 0.3027 |
| Labeler 2, Labeler 1 | –0.2379 | –0.0271 | 0.1876 | –0.4442 |
| Labeler 4, Labeler 1 | 0.1188 | –0.0034 | 0.2723 | –0.2347 |
| Labeler 3, Labeler 2, Labeler 4 | 0.0409 | 0.0955 | 0.2832 | 0.0879 |
| Labeler 3, Labeler 2, Labeler 1 | –0.0077 | 0.0786 | 0.3085 | –0.0626 |
| Labeler 3, Labeler 4, Labeler 1 | 0.1540 | 0.0001 | 0.2293 | 0.0342 |
| Labeler 2, Labeler 4, Labeler 1 | –0.0383 | –0.0050 | 0.2122 | –0.1720 |
| All | 0.0586 | 0.0576 | 0.2694 | –0.0098 |
