# Supplementary material for: Objectively Quantifying Pediatric Psychiatric Severity Using Artificial Intelligence, Voice Recognition Technology, and Universal Emotions: Pilot Study for Artificial Intelligence-Enabled Innovation to Address Youth Mental Health Crisis
Source: JMIR Res Protoc. 2023 Oct 23;12:e51912. doi: 10.2196/51912 (PMC10628686; doi:10.2196/51912)
Supplement: Multimedia Appendix 6 [file resprot_v12i1e51912_app6.pdf]

Proposal:1938206 **PI Name:**Alemu, Yared

INSTITUTION: TQIntelligence, Inc.
NSF PROGRAM: SBIR Phase I
PROPOSAL TITLE: SBIR Phase I: Automated Emotional Distress Severity Classification Using Speech Analytics and SFSS for SUD and OUD-Related ACE and Trauma

PANEL SUMMARY:
Panel Summary

What is the intellectual merit of the proposed activity?

The goal of this SBIR project is to develop an automated way to detect emotional distress and functional impairments in children and adolescents. The PIs will leverage a software app called TQI to collect voice samples and analyze them using ML techniques to detect these distress signals.

What are the broader impacts of the proposed activity?

This proposal will detect and predict mental health disorder severity of at least 2 million American children and adolescents whose parents have OUD each year, with the capability of providing feedback at the point of care for patients, families, and mental health providers.

What is the commercial potential of the proposed project?

The proposal has strong potential for commercial impact as they can charge a small fee to insurance companies to avoid significant costs of care at a later stage in the life of the children.

Strengths:

+ The idea of extracting distress signals using voice samples is quite interesting.

+ The availability of a channel for collecting data using the TQApp is a big strength.

+ Close ties to orgs (with support letters) such as Family Ties, Georgia Hope provides them adequate patients to collect appropriate data to carry out the proposed work.

+ The team seems to be quite strong to carry out the work.

+ The broader impact of this project is huge as it reaches to the most vulnerable section of population and can lead to significant societal benefits as today's children are tomorrow's adults.

Weaknesses:

- The proposed approach of using Gaussian Mixture Model, EM, and RNNs was presented but no justification was given as to why they expect these approaches will be accurate.

- There is little preliminary analysis of the data to suggest that this architecture will actually work.

Suggestions: None

The panel assigned the following overall ranking to this proposal: Competitive

The summary was read by/to the panel and the panel concurred that the summary accurately reflects the panel discussion.

Title:SBIR Phase I: Automated Emotional Distress Severity Classification Using Speech Analytics and SFSS for SUD and OUD-Related ACE and Trauma

Institution:TQIntelligence, Inc.

NSF Program:SBIR Phase I

Principal Investigator: Alemu, Yared

Rating: Fair

Review:

In the context of the five review elements, please
evaluate the strengths and weaknesses of the proposal with respect to intellectual merit.

This SBIR Phase I project will develop a voice-based tool to classify emotional distress severity for children and adolescents (families with low socio-economic status) at-risk of trauma due to Adverse Childhood Experiences. The research work will focus on testing and validating two machine learning models to identify and predict emotional disorder severity.

Strength:
+ Emotion analysis fro voice is an interesting research area for machine learning.

Weaknesses:
- The major research and innovation of this project is to use machine learning to classify emotional disorder severity from voice, which is task 1.4. However, only a very small part of the proposal description is spent on discussing the technical approach. Many problems and concerns are not addressed, e.g., any existing methods? why will the proposed method work? The Gaussian Mixture Model and LSTM are mainly used in speech recognition (sequence data), which convert speech to text. Why will it work on detecting emotion?

- As the main innovation and risk is on developing machine learning algorithms, it is disappointing and confusing why so much discussion and budget is on data collection, therapist training, and personnel with general management and startup experience.

- Different from speech recognition, personal talking style can affect an automatic emotion identification tool significantly. Personalization should be a factor in building such a tool.

In the context of the five review elements, please
evaluate the strengths and weaknesses of the proposal with respect to broader impacts.

Strength:
+ The proposed work will have positive impact to society.
+ The project has recruited two Georgia Based Behavioral Health Organizations as Pilot sites for product development.

Weaknesses:
- The commercialization and marketing plan is lack of details and not convincing.
- It is disappointing and unrealistic to focus hiring on sales while a solid product is still under development.
- The CVs do not follow NSF requirements.

Please evaluate the strengths and
weaknesses of the proposal with respect to any additional solicitation-specific review criteria, if applicable

Summary Statement

The proposed work has positive impact to health and society in general. However, there are serious flaws with technical discussion, project plan and priority setting, innovation, personnel, and budget in this project.

Title:SBIR Phase I: Automated Emotional Distress Severity Classification Using Speech Analytics and SFSS for SUD and OUD-Related ACE and Trauma

Institution:TQIntelligence, Inc.

NSF Program:SBIR Phase I

Principal Investigator: Alemu, Yared

Rating: Excellent

Review:

In the context of the five review elements, please
evaluate the strengths and weaknesses of the proposal with respect to intellectual merit.

The goal of the project is to develop an automated way to detect emotional distress and functional impairments in children and adolescents. The PIs will leverage a software app called TQI App that has already been developed to collect the data, and analyze the voice samples using ML techniques to detect children in need and at high-risk.

Strengths:

+ The idea of extracting distress signals using voice samples is quite interesting.

+ The availability of a channel for collecting data using the TQApp is a strength.

+ Close ties to orgs (with support letters) such as Family Ties, Georgia Hope provides them adequate patients to collect appropriate data to carry out the proposed work.

+ The team seems to be extremely strong with the PI being an expert on psychological disorders with both research and implementation. It also contains seasoned business and technological leadership coupled with several experts who are leaders spanning technology and healthy data analysis.

Weaknesses:

- The proposed approach of using Gaussinan Mixture Model, EM, and RNNs was presented but no justification was given as to why they expect these approaches will be accurate. There is also no preliminary analysis of the data to suggest that this architecture will actually work.

In the context of the five review elements, please

evaluate the strengths and weaknesses of the proposal with respect to broader impacts.

The project has the potential to detect and provide care to the highly vulnerable (and large) population of children of substance-abuse parents, that are often prone to mental and psychological disorders if left untreated. The technological advances made by this project will also help address the shortage of trained mental health professionals in providing mental care to families etc.

Please evaluate the strengths and
weaknesses of the proposal with respect to any additional solicitation-specific review criteria, if applicable

Summary Statement

I really liked the proposal. They have identified a nice problem that can be potentially solved with ML/AI techniques. The team is also extremely strong. They also have strong ties with organizations to access data required to carry out the project. The only remaining risk is whether the proposed technology solution for analysing voice samples will be accurate, which I think is worth funding.

Title:SBIR Phase I: Automated Emotional Distress Severity Classification Using Speech Analytics and SFSS for SUD and OUD-Related ACE and Trauma

Institution:TQIntelligence, Inc.

NSF Program:SBIR Phase I

Principal Investigator: Alemu, Yared

Rating: Good

Review:

In the context of the five review elements, please
evaluate the strengths and weaknesses of the proposal with respect to intellectual merit.

Strength:
1. This SBIR project proposes to develop a voice-based emotional distress severity classification tool for children and adolescents at-risk of trauma due to Adverse Childhood Experiences (ACE).
2. Their tool will help mental health providers to detect the severity of emotional distress early, efficiently, and automatically.

In the context of the five review elements, please
evaluate the strengths and weaknesses of the proposal with respect to broader impacts.

1. The proposal will improve the timely and objective measurement of mental health issues and vulnerabilities in children and adolescents using innovative technology.
2. The proposal will divert high-risk youth from joining the next cohort of a population with a deadly Opioid Use Disorder.

Please evaluate the strengths and
weaknesses of the proposal with respect to any additional solicitation-specific review criteria, if applicable

This proposal will detect and predict mental health disorder severity of at least 2 million American children and adolescents whose parents with OUD each year, with the capability of providing feedback at the point of care for patients, families, and mental health providers.

Summary Statement

In this proposal, the PIs plan to develop a voice-based emotional distress severity classification tool for children and adolescents at-risk of trauma due to Adverse Childhood Experiences (ACE). This proposal will help 2 million American children and adolescents whose parents with OUD each year. The tool can also help mental health providers to detect the severity of emotional distress early, efficiently, and automatically. I have a little concern on their experience of development of deep learning based

method.

Title:SBIR Phase I: Automated Emotional Distress Severity Classification Using Speech Analytics and SFSS for SUD and OUD-Related ACE and Trauma

Institution:TQIntelligence, Inc.

NSF Program:SBIR Phase I

Principal Investigator: Alemu, Yared

Rating: Fair

Review:

In the context of the five review elements, please
evaluate the strengths and weaknesses of the proposal with respect to intellectual merit.

The investigators propose to develop a machine learning algorithm that detects clinically relevant emotional distress in speech samples from at-risk youth receiving mental health and family preservation services. The algorithm to detect and predict emotional disorder severity is likely to be preliminary and may require additional data and funding to develop a more robust classification system.

+ Benchmarks and evaluation metrics are included.

- The innovation appears to be modest and is not clearly articulated. There is a great deal of prior work on emotion detection that appears to be overlooked.

- The approach in general is not rigorous.

- The PI has limited publications and research track records. The speech processing expertise appears to lie outside the company.

- The bio-sketches should be in NSF format.

In the context of the five review elements, please
evaluate the strengths and weaknesses of the proposal with respect to broader impacts.

The proposed study, if successfully executed, will have a broader impact on community health and families.

The broader impacts on other disciplines is not highlighted.

Please evaluate the strengths and
weaknesses of the proposal with respect to any additional solicitation-specific review criteria, if applicable

- It is not clear how the revenue is calculated.

- The customer validation could be further expanded.

Summary Statement

The investigation is timely and significant. A number of concerns including limited innovation, challenges with approach and the limited research experience of the team of investigators whose primary appointments reside with the company limit the enthusiasm of the reviewer.
